# IUC26664-89 De-escalated management of stage I seminoma: three decades of real-world outcomes

**DOI:** 10.1093/oncolo/oyag286.030

**Published:** 2026-08-21

**Authors:** M P Juan Gonzalez, C Martinez Martinez, A J Montalban Valverde, P A Barranzuela Decoll, Y El Farh Boumajdil, E J Soria Hernandez, J A Mendez Garcia, E Adrover Cebrian, A Fernandez Aramburo

**Affiliations:** Complejo Hospitalario Universitario de Albacete - Spain; Complejo Hospitalario Universitario de Albacete - Spain; Complejo Hospitalario Universitario de Albacete - Spain; Complejo Hospitalario Universitario de Albacete - Spain; Complejo Hospitalario Universitario de Albacete - Spain; Complejo Hospitalario Universitario de Albacete - Spain; Complejo Hospitalario Universitario de Albacete - Spain; Complejo Hospitalario Universitario de Albacete - Spain; Complejo Hospitalario Universitario de Albacete - Spain

## Abstract

**Background:**

To evaluate long-term outcomes, relapse patterns and clinicopathological factors associated with recurrence in patients with clinical stage I seminoma managed in routine practice over three decades.

**Methods:**

We performed a retrospective single-centre study including patients diagnosed with clinical stage I seminoma between 1993 and 2025. Baseline clinicopathological features, treatment strategy after orchiectomy, relapse events and relapse timing were collected. Post-orchiectomy management was classified as active surveillance or adjuvant carboplatin. Associations between clinicopathological variables and relapse were explored using descriptive statistics and Cramér’s V.

**Results:**

A total of 113 patients were included. Median age at diagnosis was 36 years. Overall, 21.2% had stage IB disease. Tumour size was greater than 4 cm in 56.0% of patients, rete testis invasion was present in 26.5%, and lymphovascular invasion was identified in 16.8%. After orchiectomy, 59.3% of patients were managed with active surveillance and 39.8% received adjuvant carboplatin. Six relapses were observed, corresponding to an overall relapse rate of 5.3%. All relapses occurred in patients initially managed with surveillance. Mean time to relapse was 17.2 months. Among the evaluated pathological factors, lymphovascular invasion showed the strongest association with relapse (Cramér’s V 0.30; p  =  0.006), whereas the absolute number of events limited further multivariable analysis.

**Conclusions:**

In this real-world cohort of clinical stage I seminoma, relapse rates were low and all recurrences occurred after surveillance, supporting the overall safety of a de-escalated post-orchiectomy strategy when combined with structured follow-up. Lymphovascular invasion emerged as a potential marker of relapse risk in this dataset, although the small number of events precludes definitive conclusions. These findings support individualized counselling and highlight the value of long-term real-world series to refine risk-adapted management in stage I seminoma.

